# Evolving intellectual property landscape for AI-driven innovations in the biomedical sector: opportunities in stable IP regime for shared success

**DOI:** 10.3389/frai.2024.1372161

**Published:** 2024-09-17

**Authors:** Abhijit Poddar, S. R. Rao

**Affiliations:** ^1^Centre for Bio-Policy Research, MGM Advanced Research Institute, Sri Balaji Vidyapeeth (Deemed-to-be-University), Bahour, Pondicherry, India; ^2^Sri Balaji Vidyapeeth (Deemed-to-be-University), Bahour, Pondicherry, India

**Keywords:** artificial intelligence, intellectual property, biomedical sector, AI inventorship, copyright, patent, laws

## Abstract

Artificial Intelligence (AI) has revolutionized the biomedical sector in advanced diagnosis, treatment, and personalized medicine. While these AI-driven innovations promise vast benefits for patients and service providers, they also raise complex intellectual property (IP) challenges due to the inherent nature of AI technology. In this review, we discussed the multifaceted impact of AI on IP within the biomedical sector, exploring implications in areas like drug research and discovery, personalized medicine, and medical diagnostics. We dissect critical issues surrounding AI inventorship, patent and copyright protection for AI-generated works, data ownership, and licensing. To provide context, we analyzed the current IP legislative landscape in the United States, EU, China, and India, highlighting convergences, divergences, and precedent-setting cases relevant to the biomedical sector. Recognizing the need for harmonization, we reviewed current developments and discussed a way forward. We advocate for a collaborative approach, convening policymakers, clinicians, researchers, industry players, legal professionals, and patient advocates to navigate this dynamic landscape. It will create a stable IP regime and unlock the full potential of AI for enhanced healthcare delivery and improved patient outcomes.

## 1 Introduction

Artificial Intelligence (AI) refers to human-created intelligent systems designed to outperform their predecessors in efficiency (Ooi et al., 2023). First conceived in the 1950s, AI has grown significantly into a field integrating science and technology across various sectors. Computing, data analytics, and processing capacity advancements have fuelled this growth (Bughin et al., 2017). Today, AI has proved its mettle in diverse arenas, including health, education, defense, finance, etc. (World Bank, 2021). Specific to healthcare, AI played a significant role in humanity’s efforts toward response and countermeasures against COVID-19. Along with frontline workers, the AI algorithm worked silently to analyze mountains of data, identify high-risk patients, optimize drug development, and guide public health interventions (OECD, 2020). Even after the abatement of COVID-19 scare, AI’s versatility has been well-accepted as a crisis-defying, resilience-forging force. Predictive modeling and disease forecasting fuelled by AI’s data-driven insights have surpassed human capabilities. AI-based diagnostics have increased detection efficiency and accuracy and supported technology deployment in resource-poor settings. AI has expanded the scope for rapid screening of novel drug targets and has reduced the development time, attrition rate, and drug and vaccine discovery costs. Likewise, AI has potentially fast-tracked the concept of personalized medicine (Schork, 2019). It improved treatment plans, predicting patient outcomes, and identifying individuals at risk of diseases.

These spectacular developments prompted innovators to continue R&D, evolve, and fine-tune AI innovations. The digital health sector witnessed an expansion of the market through AI. The United States witnessed a drastic rise in digital health start-ups that secured over 700 funding deals, crossing over 29 billion in 2021 (Miles, 2022). The rise is proportional to increased public and private sector investment in AI-based solutions in the biomedical sectors. Funding for AI-based research projects in the healthcare sector has been the largest compared to other sectors (Buch et al., 2018). In 2022, total investment rose to 6.1 billion in the biomedical sector (Stanford, 2023). As estimated, the current AI-based healthcare market stands at 22.45 billion. It will grow exponentially with a CAGR of 36.4% in the next 5-6 years (Grand View Research, 2022). The extensive utility of AI in the COVID-19 pandemic also triggered high hopes for novel biomedical innovations to support the management of future inadvertent biological threats.

With accelerated innovations, intellectual property protection became important. Unlike the concept of ‘publish or perish’ for most scientific innovations of the pre-AI era, AI innovators and innovations are more inclined toward IP protection, especially for its translational capacity and significant commercial interests in a competitive landscape. However, in a traditional human intelligence-centric IP regime, accommodating the intellect of the artificial brain is a challenge. It sometimes conflicts with the fundamental definition of the creator of an intellect. Rapidly navigating this challenging IP landscape is essential for the AI-driven biomedical sector. Otherwise, legal uncertainty will hinder innovation and can delay the translation of bench-to-bedside technology and broader adoption for improved and resilient healthcare (Morris et al., 2011). It may also raise conflicting IP rights and harm data privacy. In this review, we discussed these major IP-related challenges in the patent and copyright sector by citing the legal instruments of a few nations like the United States, the EU, China, and India. We further identified the scope and opportunities for removing these hurdles to enable a stable IP-driven AI ecosystem.

## 2 Major IP challenges

### 2.1 Attribution and inventorship: when machines create, who owns the idea?

Through AI, a machine can be trained to conceive ideas and create innovations independently. Human interventions are only restricted to implementing a suitable AI algorithm wherein the machine evolves with the assigned task. As such, determining who owns an AI-generated invention or creation often becomes a tangled web(Hutson, 2023). Is it the programmer who designed the AI algorithms, the person who provided the training data, or the AI itself? In applying existing IP laws, especially patents and copyrights, it is challenging to assign ownership, leading to potential disputes and hindering the incentivizing of further innovation.

Additionally, many AI algorithms operate like “black boxes.” It makes it difficult to understand their internal workflow and how they arrive at their outputs. This opacity poses challenges in proving originality and defending against infringement claims, as it might be unclear whether an AI-generated work infringed on existing works or vice versa. The situation can be well articulated by citing the cases on the novel drug discovery platforms powered by AI algorithms (Moingeon et al., 2022). AI innovators develop AI modules and train them with massive datasets, including genetic, chemical, toxicological, drug efficacies, performances, etc. The trained AI model runs complex operations to autonomously identify promising drug targets and candidates by analyzing millions of datasets and selectively detecting complex patterns that are undetectable by humans. This provides the innovators with a basket of novel molecules that can be easily verified by traditional *in vivo* and *in vitro* systems. A few can become successful drugs and capture the market following clinical trials and other regulatory norms.

The landscape of drug discovery is undergoing a dramatic transformation fueled by the power of artificial intelligence. SyntheMol, a groundbreaking generative AI model, leverages a novel approach to accelerate the discovery of antibiotics (Swanson et al., 2024). It utilizes a generative adversarial network (GAN), essentially pitting two neural networks against each other. One network, the generator, creates new molecular structures, while the other, the discriminator, evaluates their realism and similarity to existing drugs. Through this competitive process, SyntheMol evolves to generate chemically feasible, drug-like molecules with the potential to combat various infections. This approach has been validated empirically, demonstrating the power of AI in designing innovative and potentially life-saving antibiotic candidates. Likewise, DeepPurpose stands out as a versatile deep learning platform offering a one-stop shop for drug discovery tasks (Huang et al., 2021). Its user-friendly interface allows researchers to train and deploy models for predicting drug-target interactions, a critical step in identifying potential drug candidates. DeepPurpose’s strength lies in its customizability, offering a vast array of encoders for compounds and proteins, enabling researchers to tailor models to specific needs. Beyond DTI prediction, DeepPurpose empowers researchers with functionalities for predicting drug and protein properties, analyzing drug–drug interactions, and even protein–protein interactions (Nguyen et al., 2021). This comprehensive approach, coupled with its ability to integrate public datasets and streamline workflows, significantly accelerates drug discovery by facilitating the rapid identification of promising drug candidates. Drug repurposing offers a compelling strategy to expedite drug development by discovering new applications for existing medications. Predicting drug-target interactions is paramount to this process. However, traditional models often rely on simplified representations of drug molecules. GraphDTA introduces a novel approach, representing drugs as graph structures, and leverages the power of graph neural networks. This innovative approach significantly outperforms existing methods in predicting drug-target affinity, highlighting the value of graph-based representations for modeling complex molecular interactions.

Given these developments and to place it under IP protection, the first puzzle is determining who deserves credit for the invention (i.e., the successful drug). The human researcher has not conceived the core innovation driven by AI. Instead, it is the AI that plays a crucial role in conception by identifying previously unknown candidates. It creates conflicts where, traditionally, inventorship is awarded to individuals who contribute to the “conception” of the invention, the “reduction to practice,” and the “non-obviousness” of the invention. These uncertainties hinder innovation as developers face difficulties securing patents and attracting investment.

### 2.2 Copyright quandaries: protecting the creativity of machines?

Another form of IP is copyright, which provides the creator legal rights over their literary and artistic works to protect an individual’s creativity (WIPO, 2023). The biomedical sector is one of the most significant breeding grounds for copyright content, including written materials, databases, coding, medical illustrations, images, audiovisual materials, etc. While copyright gives credit to the developer, it also prevents unauthorized appropriation of their findings. However, unlike patents, copyright protection is not absolute. Rather, specific fair use, like research, teaching, etc., allows limited use of copyright. The recent open-access scientific journals grant Creative Commons license, allowing unrestricted reuse with proper attribution. This strikes a delicate balance between promoting open knowledge dissemination and ensuring recognition for the researchers’ efforts.

Following the advent of AI, the realm of creativity is no longer solely the domain of humans. However, the origin of literary and artistic works, the highest form of creativity, was considered a defining characteristic of humans (Morriss-Kay, 2010). In fact, modern human creativity lies in genetic networks containing more than 200 non-protein-coding genes and their impacts on other gene functions (Zwir et al., 2022). AI rippled it with its creative outputs. It has genuinely been an expansion of creativity from the genetic network (human creativity) to the digital code network (AI’s creativity). AI has created fascinating artwork, designs, and documents. Projects like “Deep Dream” and “GANs” (Generative Adversarial Networks) utilize machine learning to create abstract landscapes, surreal portraits, and even eerily realistic renditions of existing art styles. AIs enabled creative outputs like novel proteins, drug designs, and even scientific papers in biomedical science.

The *de novo* protein design sector has been revolutionized with multiple AI-driven tools. For example, AlphaFold, developed on the 14^th^ Critical Assessment of Protein Structure Prediction (CASP14), utilizes physical, biological, and sequence knowledge to design highly complex and ‘never-before-existing’ proteins (Jumper et al., 2021). It can model highly complex proteins like intertwined homomers and proteins folded around unknown haem groups. It has found tremendous applications in molecular replacement, cryogenic electron microscopy maps, human proteome analysis, etc. Likewise, Baker Lab developed RoseTTAFold, a three-track” neural network-based AI model that only takes 10 min to develop new protein structures, poorly understood protein structures with minimal information input (Baek et al., 2021). These successes have enabled AI-based protein design start-ups like Profuent, Arzeda, Cradle, Monod Bio, etc., to raise business funding (Eisenstein, 2023). As part of an AI-driven novel drug development pipeline, AI can design drugs, perform synthesis, and optimize them against selected targets. Such *de novo* design has become widespread after the introduction of AI-implemented generative adversarial networks (GAN), recursive neural networks (RNN), etc., which have shown promise in generating novel drugs. deepDTnet is an AI algorithm combining chemical, genomic, phenotypic, and cellular networks for drug design (Moingeon et al., 2022). Likewise, MolAICal AI, with its deep learning module, can design 3D drugs with good binding scores in target protein pockets (Bai et al., 2021). These AI-driven creativities are inspiring and a great way to design novel drugs that are extremely difficult by simple human interventions. Also, it reduces the time to design and therefore bears a greater chance for the lowest time to market.

AI’s creativity is becoming undeniable in literary creations, too. AI’s ability to write content has vastly accelerated scientific publications (Hosseini et al., 2023). Before these AI, computer programs largely replaced paper-based writing yet could not create content alone. However, neural language processing generative AI models like ChatGPT, Meta’s Galactica, Google’s Bard, etc., can create complete content suitable for publication. Although most of these generative models are free and do not advertise their capacity to write a research article, AI tools like Jenni AI, HyperWrite, Paperpal, etc., claim to do so with exciting pricing packages. These tools are increasingly becoming attractive across academia for their capacity to remarkably reduce human labor and time in writing competitive grants and scientific manuscripts (Tay, 2021). Current world science practice evolves around competition for funding and quantity of publications as metrics for assessing investigator professional careers where supportive roles of these tools are undeniable. Although critics are raising concerns about research ethics, quality, and scientific integrity while preparing AI-based manuscripts, one can ask what the harm is to using AI for this when the entire S&T becomes a competitive model. Why do nations push for publication quantity for career growth instead of allowing deeper and quality research? Why should researchers restrict AI’s use in writing competitive grant applications for research funding that is extensively time-consuming and does not necessarily provide funding? In contrast, AI content could make a more precise pitch against the call for proposals by funding agencies and significantly increase the chance of securing funding. It can even enable researchers to apply for many grants while saving time for actual research work. Nevertheless, AI will prevail in literacy work and improve further as time goes on. Realizing this, some publication houses, like JAMA, Elsevier, etc., have issued guidelines for the responsible use of AI in their work for submission (Flanagin et al., 2023).

All such creative works pose a considerable copyright challenge. A prudent question arises: Are these genuinely creative, or are ‘copies’ or ‘imitations’ of available information and using those in a predefined machine training architecture? Furthermore, the intersection of innovation and copyright in AI-driven creation gives rise to complex ethical dilemmas. A salient example is the development of polygenic risk scores, which leverage AI to predict disease susceptibility based on genetic data. While promising, these scores are often trained on datasets predominantly of European population, raising concerns about algorithmic bias and potential inequities in healthcare (Popejoy and Fullerton, 2016). This phenomenon underscores a broader challenge: the overreliance on data from Western, Educated, Industrialized, Rich, and Democratic (WEIRD) populations in various research fields (Norori et al., 2021). As a result, AI models trained on such data may perpetuate existing biases, leading to inaccurate or harmful outcomes. The implications for accountability are profound. Assigning responsibility for AI-generated outputs that cause harm—whether to the creator, the AI system itself, or the AI developer—presents a significant legal and ethical challenge.

Addressing these ethical challenges necessitates a multifaceted approach. Establishing clear ethical guidelines, ensuring transparency, and updating intellectual property frameworks are essential steps. Zou and Schiebinger proposed a dual-pronged approach to addressing the ethical challenges posed by AI (Zou and Schiebinger, 2021). To mitigate immediate risks, they advocated for enhanced post-deployment monitoring of medical AI systems. For a more sustainable solution, they emphasized the integration of social, cultural, and ethical considerations into AI curricula. This long-term strategy aims to cultivate a new generation of AI developers equipped to build responsible systems.

Further, creativity shows humanity’s free will and thought. Till now, it has essentially been absent in current AI models. As such, can the AI design be marked as creative or simply channeling some datasets for the task assigned by the creator? Even though the above are considered part of creativity, most nations like the United States, Germany, Spain, etc., do not entertain non-human creativity as copyright. Alternatively, nations like India, the United Kingdom, and New Zealand delegate the copyright to the human developer but not to the AI or its end users who use it to create creative content (Guadamuz, 2017). This ambiguity creates risks for creators and hinders knowledge sharing, potentially hampering progress in critical areas like healthcare. Not precisely in the biomedical sector, yet several lawsuits involving AI and copyright have also turmoiled the existing grey areas (Samuelson, 2023). Meaningful resolutions are far-reaching and add to the complexities.

### 2.3 Data dilemmas: expanding challenges in a tricky triangle

The biomedical sector in research to healthcare produces a large amount of data. Data without patient attributes, like genomics, proteomics, transcriptomics, etc., are generated and mainly deposited in public databases for R&D purposes. Other sensitive data having patient identifiers include medical imaging, patient records, disease and epidemiological data, immunological data, etc. These are essentially the output from healthcare settings and are mainly used for clinical decision-making. These sensitive data are also used for medical research following necessary ethical clearances. Interpreting these large databases by combining different datasets can lead to remarkable findings, including but not limited to disease forecasting, personalized medicine, etc. Exactly here, AI became popular and has outperformed human intelligence for its ability to deal seamlessly with extensive data and find rationale links between them. However, the success of AI models depends on the quality and quantity of data. The more it trains with unbiased data, the more desirable output it can provide for significant medical benefits.

In biomedical innovation, the amount of data needed to train an AI model depends on several factors, often exceeding the requirements of other application areas. However, there are no specific estimations possible. Medical image analysis generally requires an average of thousands to millions of labeled images depending on task complexities like disease detection, organ segmentation, etc. AI needs almost millions of individual genomes or exomes data to identify disease-associated genes or predict treatment responses. Likewise, millions to billions of patient records datasets are needed to train AI models for disease forecasting, understanding population health trends, predicting disease risks, and developing a personalized treatment plan. The pre-AI era has never witnessed such high demand for data analysis. Biomedical innovation by human innovators required only a fraction of the current size of data required for AI-based analysis. It has spotlighted biomedical data sources and reinstated the three intricate tricky issues, i.e., ownership, sharing, and privacy of biomedical data, leading to extraordinary debates and deliberations. Such debates also exposed data safety, security, and ethics challenges with an overarching influence on IP rights and their protection (Minssen and Pierce, 2018). Unless the developer declares the source and associated IPs with the million datasets used for AI training, it is practically impossible to determine cases of IP infringements. Even for the black box paradigm, it is challenging to determine if AI has taken IP-protected data and if proper acknowledgment is made for such.

## 3 AI-focused IP legislative landscape: The laws of the land

### 3.1 United States IP framework

The Patent Act (Title 35 of the United States Code) of the United States empowers the United States Patent and Trademark Office (the USPTO) to grant patents for new, useful, novel, and non-obvious innovations. Under the legal regime, AI innovations have faced hurdles in claiming patent protection. There have been discussions on determining the eligibility of AI-based innovations for patents. As the first in the world, the USPTO held an IP policy discussion conference in 2019. It connected stakeholders of AI innovation to promote AI–IP understandings and how the United States Patent Act is flexible enough to accommodate these newer fields (USPTO, 2020). It was followed by a request for comments on patenting AI innovations. Stakeholders agreed on the patentability of AI innovations as a tool for incentivization and further encouragement in the field. Also, they noted that United States laws are flexible to accommodate AI innovations. It reflects well in the United States inventorship law that can deal with AI-assisted inventions on a fact-specific case-by-case basis. Consequently, applications for patents of AI-based inventions doubled from 2009 to 2019 and tripled the instances of patent approval. In the biomedical sector, some AI-based medical devices and processes have successfully secured patents, focusing on specific applications and improvements rather than the algorithm itself. For example, the United States Department of Health and Human Services secured a training-based automated cancer detection patent using MRI (Kwak et al., 2017). A European patent described a neural network-based model system for classifying cancer tissue as malignant or benign (Zhang and Kumar, 2010). Siemens Healthcare GmbH obtained a patent for methods and systems for AI-based medical image segmentation (Zhou et al., 2019). Ai Medical Service Inc. secured a patent for a diagnostic assistance method that uses a convolutional neural network (CNN) for disease detection by using endoscopic images of a digestive organ (Tada et al., 2021). However, the United States does not allow an AI to be an inventor for a patent. This was clarified following two innovations filed for patent by Thaler v. Vidal, citing his AI system Device for the Autonomous Bootstrapping of Unified Sentience (DABUS)” as the sole innovator. Federal Circuit rejected the application, citing that only natural persons (i.e., human beings) can be named inventors on United States patents (Thaler v. Vidal, No. 21-2347, 2022, Vidal, 2024).

United States Copyright Act of 1976 (Title 17 of the United States Code) traditionally protects “*original works of authorship fixed in a tangible medium of expression*.” However, the definition of ‘author’ is a grey area for AI innovations as United States laws do not define who can be the author. The same question as patent law also arises: is the human programmer the author, or does the AI deserve credit as the author? However, even before AI complicated the authorship issue, it seems that the United States Copyright Office only considered humans as competent authors of copyright. The first such evidence was from the denial of authorship to monkeys that took photos (Reuters, 2018). It was later confirmed during 2022-2023 after the Copyright Office canceled the copyright for AI-generated graphics, stating that AI authored the visual material (Zirpoli, 2023). The office released guidance stating that AI-generated output cannot be considered for human authorship. United States Copyright Office, however, continues to receive many applications for copyright that generative AI models have produced. To clarify the copyright regime during the AI era, the United States copyright office has taken several stakeholder-focused initiatives, including the Notice of Inquiry and Request for Comments (“RFC”) (United States Copyright Office, Library of Congress, 2023). It is expected that the RFC will enable the United States Copyright Office to determine the need for further legislative or regulatory steps and transparency and disclosure in AI copyright work.

The United States ranks first in the, 2023 Intellectual Property Index (United States Chamber of Commerce’s Global Innovation Policy Center, 2023). This denotes proactive innovation and the IP ecosystem that significantly builds the nation’s resilience in this direction. The trend continues as the United States looks to build a robust and progressive AI ecosystem while addressing the existing practice and policy challenges, including IP. As such, the White House Office of S&T Policy issued a legally non-binding white paper to support the overall development of policies that protect civil rights and promote AI building, deployment, and governance (White House Office of Science and Technology Policy, 2022).

In this direction, the United States National Artificial Intelligence R&D Strategic Plan 2023 advances previous efforts (i.e., the 2016 and 2019 national AI R&D strategic plans) by providing a roadmap to sustain American leadership in AI. It identifies critical research areas, aligns federal resources, and prioritizes the development of reliable AI systems (National Science and Technology Council, 2023). It outlines nine key strategies to maintain America’s AI leadership. These include long-term investments in fundamental AI research, fostering human-AI collaboration, addressing ethical and societal implications, ensuring AI safety and security, building public AI datasets, developing evaluation standards, understanding AI workforce needs, strengthening public-private partnerships, and establishing a principled approach to international AI cooperation. The plan emphasizes responsible innovation, public good, and addressing challenges like climate change and healthcare through AI.

To oversee and implement a comprehensive strategy for AI research and development, the National Artificial Intelligence Initiative (NAII) Act was enacted in 2021. The legislation mandates coordination among federal agencies, including the Department of Defense, civilian departments, and intelligence entities, to align AI research efforts. As a central hub for federal coordination, the National AI Initiative Office was established in January 2021. This office facilitates collaboration among government agencies, the private sector, academia, and other stakeholders in AI research, development, and demonstration. Through regular public outreach, it promotes the dissemination of AI technologies, innovations, and best practices across the federal government. Similarly, the National Institute of Standards and Technology (NIST) was tasked with developing voluntary standards for reliability and safety of AI systems. In response to this mandate, NIST developed the AI Risk Management Framework (AI RMF) (NIST, 2024a). This framework, established through extensive public engagement, provides a structured approach to managing AI-related risks throughout the system’s lifecycle. Released in January, 2023, the AI RMF emphasizes the importance of trustworthiness by incorporating relevant considerations into AI design, development, deployment, and evaluation. To facilitate implementation, NIST has complemented the framework with additional resources, including a playbook, roadmap, and crosswalk. Moreover, the Trustworthy and Responsible AI Resource Center supports the adoption and global alignment of the AI RMF. Furthermore, Recognizing the unique challenges posed by generative AI, NIST released a specific profile for this technology in July 2024 (NIST, 2024b). This profile offers tailored guidance for managing risks associated with generative AI systems.

The recently enacted Future of AI Innovation (FAII) Act of 2024 represents a significant advancement in U.S. AI policy (Senate - Commerce, Science, and Transportation, 2024). Building upon the NAII Act, the FAII act adopts a more comprehensive approach, prioritizing industry collaboration, global leadership, and standardized evaluation. It adopts a holistic approach including research, development, deployment, and ethical considerations. Unlike the NAII act, which primarily focused on coordinating research, the FAII act places a strong emphasis on developing standardized metrics and evaluation tools for AI systems. This ensures that AI development is grounded in rigorous evaluation, leading to more reliable and trustworthy systems. The FAII act goes beyond government-led initiatives by actively promoting innovation within the private sector. It provides incentives and support for AI companies of all sizes, fostering a robust and competitive AI ecosystem. The FAII Act recognizes the global nature of AI development and emphasizes international cooperation. It seeks to establish the United States as a leader in setting global AI standards and norms, ensuring that American values and interests are at the forefront of international AI governance. This comprehensive framework ensures that the United States is prepared to address the full spectrum of challenges and opportunities presented by AI.

Even before the AI regime, the United States legal landscape governing data ownership and privacy in the biomedical sector has been a complex interplay of federal and state regulations. The Health Insurance Portability and Accountability Act (HIPAA) primarily safeguards patient health information (PHI) held by covered entities, imposing security and privacy standards. It establishes national standards for safeguarding medical records and other personal health data. Key provisions include defining what constitutes PHI (including names, addresses, birth dates, Social Security numbers, and medical records), setting strict rules for how PHI can be used and disclosed, granting patients specific rights over their health information, and imposing penalties for violations. HIPAA also mandates robust security measures to protect electronic health information (e-PHI) from unauthorized access, use, or disclosure. However, the breadth of biomedical data extends beyond PHI, necessitating the involvement of other legal frameworks. The Federal Trade Commission (FTC) Act, though centered on consumer protection, has been instrumental in enforcing data privacy and security obligations within the sector. The increasing adoption of comprehensive state privacy laws further complicates the regulatory environment, requiring organizations to navigate a patchwork of requirements. Beyond privacy, the Common Rule governs human subjects research, ensuring ethical conduct and participant protection. As the biomedical field advances, particularly with technologies like artificial intelligence and machine learning, challenges in data ownership, privacy, and security intensify. Determining who owns biomedical data, especially genetic information and patient-generated health data, is a complex legal and ethical issue. Safeguarding sensitive data from breaches while enabling innovation demands a robust legal and operational framework, including data governance policies, privacy impact assessments, and stringent security measures.

The advent of artificial intelligence (AI) has significantly challenged the existing legal framework for data ownership and privacy in the biomedical sector. The United States government has recognized the implications of AI on privacy and has taken initial steps to address these challenges. The White House Executive Order on AI, issued in October 2022, represents a significant step toward establishing a regulatory framework for artificial intelligence in the United States (White House, 2023). While the order encompasses a broad range of AI-related issues, it has specific implications for data privacy and security in the biomedical sector. The order directs federal agencies to develop risk management frameworks for AI systems, including those used in healthcare. This includes assessing potential risks to privacy, civil rights, and national security. Additionally, the order emphasizes the importance of data privacy and security, calling for agencies to prioritize data protection measures when developing or acquiring AI systems. A key focus of the order is promoting responsible innovation. It encourages the development of AI systems that are safe, effective, trustworthy, and lawful. For the biomedical sector, this implies a need for AI systems that respect patient privacy, maintain data security, and avoid biases that could lead to discriminatory outcomes (Daniel, 2024). While the Executive Order provides a foundational framework, it is not a comprehensive regulatory regime. It sets out broad principles and directives for federal agencies, but specific regulations and standards will likely require further development. Therefore, it is recognized that AI has created a ripple in existing legal regimes. However, the country proactively aligns with these changes while considering stakeholders’ views.

### 3.2 European Union IP framework

For IP being dealt by the laws of the land, the legal atmosphere in the EU, comprising 27 member states, presents a very complex labyrinth filled with unfamiliar terms and intricate procedures (Matthews and Torremans, 2023). First, there are national laws where national Patent Offices examine and grant patents independently specific to the nation. The other is EU patent law, a bundle of national patents but not a single patent. EU patent law is applied to all members and is born in the Convention on the Grant of European Patents 1973. European Patent Office (EPO) is a centralized office that implements the unitary patent system, a single application process for patent protection across most EU member states (excluding Spain and Italy). It provides cost advantages and reduces administrative burdens in the patent application process (European Commission, 2023c). EPO examines applications and grants European patents, which take effect in designated member states after translation and formal acceptance.

Unlike the United States, the EU patent is inventor neutral. It can grant patents to inventions across all fields of technology, including AI if it has patentability principles like ‘*new, involve an inventive step and are susceptible to industrial application*’ (European Patent Office, 1973, p. 52). EPO is very much evident in its view of the patentability of AI innovations by categorizing them under “computer-implemented inventions” (CII) (European Patent Office, 2022). An AI can own a patent if it applies a technical problem in a technology field and adheres to patentability principles. As such, EPO cited the case of biomedical innovation, i.e., ‘*use of a neural network in a heart-monitoring apparatus for the purpose of identifying irregular heartbeats*.’ EPO issued a revised Guidelines for Examination in, 2023 that provides rigorous methodology encompassing legal certainty and predictability for CIIs, including AI (European Patent Office, 2023). There are instances where EU patents were offered for AI for breakthroughs in healthcare. For example, a method that designs dental drilling templates using 3D scans and an artificial neural network secured an EU patent (Schneider et al., 2020). An automated diagnostic platform that can determine the health state of individuals by ML analysis of images generated by consumer computing devices was granted a patent (Dressler, 2022).

Copyright protection in the EU is essentially to harmonize standards while protecting creativity and promoting cultural diversities across the member states. As such, the EU copyright law is not one directive; instead, it contains 13 directives and two regulations issued from time to time (European Commission, 2023b). The EU copyright law covers many works, including Literary, artistic, and musical works, Films and videos, Sound recordings, Broadcasts, and Databases. Like most nations, the law grants creators and rightsholders exclusive rights, including the right to reproduce their work, communicate it to the public, distribute it, and adapt it.

At the time of writing this manuscript, copyright protection of AI-based creativity was largely a grey area in the EU. There are no legally implemented directives that specifically mention AI-based copyright issues. However, in 2019, the EU issued a Digital Single Market (DSM) directive to address digital technologies like automated computational analysis of information, i.e., text and data mining (European Union, 2019). AI and data mining are not precisely similar, but given data mining as the foundation of AI, the DSM directives, to some extent, addressed exceptions and limitations, improved licensing practices, and achieved a well-functioning marketplace for copyright. To this extent, the 2019 DSM Directive supported the EU’s growing digital economy and favored the growth of companies selling digital content.

The EU has adopted a robust legal framework for data protection, particularly in the biomedical sector (Marelli and Testa, 2018). The cornerstone of this approach is the General Data Protection Regulation (GDPR) that was introduced in 2016 but became fully enforceable on May 25, 2018. The GDPR provides a comprehensive framework for processing personal data within the EU. Key principles include lawfulness, fairness, and transparency; data minimization; accuracy; storage limitation; integrity and confidentiality; and accountability. Organizations must adhere to these principles, ensuring lawful data processing, safeguarding data subject rights, and implementing robust data protection measures.While the GDPR primarily focuses on data protection, it also indirectly influences data ownership by empowering individuals with control over their data (Starkbaum and Felt, 2019). It grants individuals substantial rights like the right to access, rectify, and erase their personal data. This effectively gives individuals a degree of control over how their data is used, processed, and stored. By placing the individual at the center of data protection, the GDPR shifts the balance of power, reducing the extent to which organizations can claim exclusive ownership over the data they collect.

The GDPR’s stringent data protection measures present a complex interplay of challenges and opportunities for AI innovation in the biomedical sector. While its emphasis on data quality and accuracy aligns with AI development needs, fostering reliable and effective models, the GDPR’s restrictions on data access can hinder AI training. Additionally, the GDPR’s focus on individual rights and fair processing promotes ethical AI development but can pose challenges for complex AI models that require extensive data and interpretability.

The EU’s Data Governance Act (DGA) and New European Innovation Agenda (NEIA) are pivotal in addressing the complexities of balancing data protection with AI innovation in the biomedical sector. The DGA specifically focuses on facilitating data sharing by introducing novel data intermediaries and encouraging data altruism (European Commission, 2023a). This can help overcome the data access challenges posed by the GDPR, providing more data for AI training while maintaining privacy safeguards. Additionally, the DGA promotes the reuse of public sector data, potentially enriching datasets for AI development. The NEIA complements the DGA by prioritizing innovation and creating a conducive environment for AI development (European Commission, 2022). It aims to foster a data-driven economy, which can accelerate AI advancements in healthcare while addressing social challenges and enabling them to reach the market. By aligning innovation policies with data governance, the NEIA can help ensure that AI development is both innovative and responsible. Together, the DGA and NEIA form a comprehensive approach to address the challenges of balancing data protection with AI innovation. They strive to create an ecosystem where data can be shared and utilized effectively for the benefit of society, while upholding the fundamental rights of individuals.

Very recently, in August 2024, the European Union enacted the Artificial Intelligence (AI) Act (Official Journal of the European Union, 2024). Positioned as the world’s first comprehensive AI legislation, the Act provides a clear regulatory environment for businesses, promotes trust in AI, and safeguards consumer interests. To stimulate innovation, the AI Act explicitly excludes AI systems developed solely for scientific research and development from its scope. However, any AI system resulting from such research must comply with the act once placed on the market. This is achieved by adopting a risk-based approach, categorizing AI systems into four tiers based on their potential impact: unacceptable risk, high-risk, limited risk, and minimal risk. While high-risk systems, such as those used in critical infrastructure or law enforcement, are subject to stringent regulations, low-risk systems face minimal requirements. A key feature of the AI Act is its commitment to safeguarding fundamental rights, including privacy and non-discrimination. The legislation also emphasizes the importance of human oversight and accountability in AI systems.

The EU AI Act has significant implications for the biomedical sector, given the high-stakes nature of AI applications in healthcare. Most AI applications in the biomedical sector are likely to be classified as “high-risk” under the AI Act. High-risk AI applications in this sector include medical diagnosis, drug discovery, and AI-integrated medical devices. This necessitates stringent compliance with regulations encompassing risk assessment, data governance, transparency, human oversight, robustness, cybersecurity, and market surveillance.

The EU AI Act, while primarily focused on regulating AI systems, has significant implications for patent law. The act’s emphasis on transparency, accountability, and the prohibition of certain AI practices could influence patent eligibility, prosecution, and enforcement. Patent offices may face challenges in determining the patentability of AI-related inventions that involve prohibited techniques or raise concerns about transparency and explainability. Additionally, the act’s requirements for data governance and human oversight could impact the scope of patent claims and the disclosure requirements in patent applications.

The intersection of the EU AI Act and copyright law presents a complex legal landscape. While the AI Act primarily focuses on regulating AI systems, it acknowledges the crucial role of data, including copyrighted material, in AI development. This raises questions about fair use, licensing, and potential copyright infringement. Moreover, the originality and authorship of AI-generated content pose challenges for copyright protection. The act also introduces transparency obligations for AI systems, potentially impacting copyright holders’ ability to monitor the use of their copyrighted material. Determining liability for copyright infringement when AI systems are involved is another complex issue.

The EU AI Act places a strong emphasis on data protection and privacy while forming a synergistic framework with DGA and NEIA. The AI act mandates stringent standards for data quality, accuracy, and reliability, while also prioritizing data minimization to reduce privacy risks. The act ensures transparency by requiring disclosures about data used to train AI systems. Additionally, it reinforces data subject rights, such as the right to access and erase personal data. These measures aim to build trust in AI technologies while safeguarding individual rights.

EU’s AI act is likely to influence the development of AI regulations in other jurisdictions by providing valuable insights and guidance for other countries and regions. The Act’s influence is evident in the growing number of countries developing their own AI regulations, often drawing inspiration from the EU’s approach (Elbashir, 2024). While challenges such as regulatory arbitrage and the complexities of global governance persist, the EU AI Act is undeniably a catalyst for international cooperation in establishing responsible AI standards.

### 3.3 India IP framework

India’s rise in innovation has been quite phenomenal. While securing the 40th rank in the, 2023 Global Innovation Index, India grew by many patents and currently occupies the 28th rank globally (Press Information Bureau, 2023). However, unlike many other countries, Indian IP legislation transitioned through many reforms and operated under the 1970s Patent Act and amendments (Lokur et al., 2023). The latest amendment, the Patents (Amendment) Act 2005, introduces a product patent regime. The Indian Act grants patents to any inventions, either new products or processes that involve inventive steps and have industrial applications. Being so old, the Act has no specific directives on patentability for AI-based innovations. The law excludes computer programs and algorithms from patents unless they have a practical application within a machine or device. Likewise, patent protection excludes diagnostic methods and therapeutic uses. These exclusion criteria create a grey area and can legally affect AI innovations, including those in the biomedical sector. Despite these, the Office of the Controller General of Patents, Designs, and Trademarks (commonly known as the Indian Patent Office) has witnessed a sharp rise in AI-based patent applications between 2002 and 2018 (Chahal et al., 2021). Such a rise was correlated with higher computing and data processing abilities and a rise in AI algorithms. In its 2021 report, NASSCOM noted that about 5,000 AI-based patents were filed in India, with 94% in the past 5 years (NASSCOM, 2024). In healthcare, M/s Niramai Health Analytix obtained an Indian Patent for AI-based innovation targeting early-stage breast cancer detection. Without clear legal instructions, IPO seems to consider the guidelines for computer-related inventions (CRIs) (last amended in 2017) to review cases involving AI innovations (Upputuri and Üner, 2023). In the best-case scenario, a specific patent amendment under the Act to address AI-based innovations will be the best option for India to clear the clouds of their patentability. Likewise, copyrighting of AI-generated work is also uncertain under India’s Copyright Act 1957 and amendments thereunder. The decades-old law delegated authorship to human beings for any literary, artistic, musical, or artistic work that is computer generated (Govt of India, 1957). To some extent, this can be used to restrict giving authorship to AI. However, contrasting evidence exists. For example, the “AI model RAGHAV” obtained co-authorship for an AI-generated artwork in 2020 (Sarkar, 2021).

Unlike the United States and the European Union, India’s legal framework for data ownership and privacy in the biomedical sector is still in its formative stages (Naithani, 2024). While the country has made strides, significant challenges persist in balancing innovation with robust data protection. India’s legal landscape is more fragmented, with the Information Technology (IT) Act, 2000 providing a foundational framework but lacking specific provisions for the biomedical sector. Later, Information Technology (Reasonable Security Practices and Procedures and Sensitive Personal Data or Information) Rules, 2011 was issued under the IT act to prevent unauthorized access to sensitive personal information, including health information. To bring consolidated approaches in data protection and ownership, several draft data protection bills were tabled in Indian parliament in 2019 and 2021. These precursor bills laid the groundwork for the final legislation The Digital Personal Data Protection (DPDP) Act, 2023 by introducing key concepts and principles related to data protection and ownership (Mukhija and Jaiswal, 2023). The DPDP Act outlines a comprehensive framework for safeguarding personal data in India. Key features include the establishment of robust individual rights such as data access, correction, and erasure. It imposes stringent obligations on data fiduciaries, requiring them to obtain valid consent, implement stringent security measures, and adhere to data minimization principles. The Act also creates a regulatory body, the Data Protection Board, to oversee compliance and address grievances. Additionally, it provides provisions for cross-border data transfers, ensuring adequate safeguards for personal data. However, the DPDP Act presents both opportunities and challenges for AI-based innovation in the biomedical sector. While the Act promotes ethical AI development, the Act’s restrictions on data processing, such as data minimization and purpose limitation, potentially limit the availability of data for AI training, potentially hindering innovation. Moreover, obtaining explicit consent for every data processing activity, as mandated by the Act, can be impractical in AI development, where data is often used for various purposes.

Despite these ambiguities, India realized AI’s transformative potential and is planning for reforms. In this direction, the NITI Aayog-the policy Advisory Body of the Federal government, published the National Strategy for Artificial Intelligence and Applications in Healthcare as a priority area for AI-based interventions (NITI Aayog, 2018). However, the report only rationalized cancer screening and treatment as the specific area of interest. It overlooked the demand landscapes in other communicable and non-communicable diseases (other genetic disorders), personalized treatments, patient monitoring, etc. The report identified that an unattractive IPR regime should be a bottleneck for AI innovation and adoption. It recommended setting up a specialized task force by the Ministry of Corporate Affairs and Government Department of Industrial Policy and Promotion (DIPP), the nodal ministry for implementing IPRs, to introduce necessary modifications to the IP regime. Almost similar recommendations came from the Parliamentary Standing Committee on Commerce in its 161^st^ report on ‘Review of the Intellectual Property Rights Regime in India.’. The committee recommended a separate category in the IPR regime to accommodate AI-based innovations with a focus on pharmaceutical research leading to drug discovery (PRS Legislative Research, 2022).

### 3.4 China IP framework

Inventions, utility models, and designs are patentable under The Patent Law of the People’s Republic of China. The law empowers the China National Intellectual Property Administration (CNIPA, formerly State Intellectual Property Office) to grant patents on satisfying novelty, inventiveness, and industrial applicability. Neither the patent law nor the patent eligibility guidelines issued by CNIPA specifically inform the patentability of AI-generated works. The 2006 Examination Guidelines have provided indirect evidence on patenting AI-related work. It specified that a simple computer program cannot be patented. However, an application where a combination of programs, software, and hardware can provide a technical solution by (i) solving technical problems, (ii) using technical measures, and (iii) producing a technical effect can be patented (Mattei et al., 2019). Therefore, AI as an application that provides technical solutions is patentable. China is now leading the world in AI-based patents. China had nearly 30,000 AI-related patents, occupying 40% of global AI patent-based applications in 2022 (Bloomberg, 2023). In the biomedical sector, China offered patents in AI innovations in medical diagnostics, nursing/caring, medical devices, data and archiving, and pharmaceuticals. In 2019, 12,325 AI patents were issued in healthcare, with the majority in the diagnostics and nursing sectors (Huateng, 2019).

In contrast to patents, China witnessed multiple court cases debating the copyright protection of AI-generated work (Wan and Lu, 2021). The cases reflect the unavailability of a precise legal roadmap on the AI work in China’s copyright law. The law grants copyright to ‘copyright owners’ who are authors, other citizens, legal entities, and other organizations (WIPO Lex, 2024). The expression author is essentially referred to as a legal Chinese citizen. As such, AI itself cannot be an author and so cannot obtain copyright. However, AI-generated work can be copyrighted to persons provided it fulfills the copyright conditions specified in the law. For example, the Chinese court denied copyright when AI only generates output despite the content’s originality (Lee, 2021). However, AI output with sufficient contribution from human intelligence was considered copyrightable (Zhuk, 2023). Following the same argument on the involvement of human intelligence, China’s court granted copyright to one AI-generated image (King and Wood Mallesons, 2023). The court cited that human intelligence set the AI model parameters that ultimately generated the final output. These decisions are markedly opposite to the major global player’s views, offering copyright to exclusive human creativity. Therefore, China’s stance on copyrightability by bimodal intelligence signified the nation’s intention to protect and incentivize AI works.

China’s approach to data ownership and privacy in the biomedical sector diverges significantly from Western paradigms (Pernot-Leplay, 2020). Prioritizing national interests over individual rights, the state exercises substantial control over data collection, use, and sharing. Data is considered a national asset, resulting in a regulatory landscape where data protection is often balanced against security concerns. This dynamic is reflected in China’s legal framework. While the Cybersecurity Law (2017) and Data Security Law (2020) provide a broad overview of data protection, the Personal Information Protection Law (PIPL) (2021) offers more specific regulations for personal data, including sensitive health information. Despite granting some individual rights, the PIPL ultimately prioritizes national interests. These regulatory nuances pose substantial challenges to AI innovation in the biomedical sector (Yao and Yang, 2023). Stringent data localization requirements, intended to safeguard sensitive information, hinder the free flow of data essential for AI training and development. Moreover, the emphasis on national security can restrict cross-border data transfer, impeding collaborations with global research institutions and access to diverse datasets.

In response, China has initiated robust efforts to regulate AI development though multiple AI regulations, and policies (Sheehan, 2023). Much emphasis has been placed on data security. The National Information Security Standardization Technical Committee’s draft specification for AI large model training underscores the nation’s commitment to building a secure and trustworthy AI ecosystem (Interesse, 2024). By outlining security measures across data annotation, pre-training, and optimized training data, this initiative aims to safeguard data privacy, prevent breaches, and enhance AI model reliability.

## 4 Discussion and way forward

AI is in great demand in the healthcare sector. Around 86% of healthcare service providers, companies, and tech vendors used AI by year 2019 (Sullivan and Schweikart, 2019). Evidence suggests more transformative benefits across the ecosystem. AI is emerging as “a fundamental tool of medicine,” transforming medical education into the more technocratic “biotechnomedical” model (Cuff and Forstag, 2023). Major transitions are in research, service, and delivery, with dominance in imaging, diagnostics, monitoring, and disease forecasting. It enhances humanity’s effort to respond better and prepare against emerging and re-emerging biothreats, natural or intentional. Many scientific publications have proof-of-concept models, devices, and technologies. In parallel, global pursuits of creating IP on AI-based innovations exist. Pharma companies witnessed a 52% rise in AI-based patent applications in, 2023. With keyword searches like ‘Artificial Intelligence’ and ‘Health’ in Patentscope, the authors obtained 2,696 patents, of which 233 are PCT (WIPO - Search International and National Patent Collections, 2024).

However, pulling all AI-based innovations across the Technology Readiness Level (TRL) levels to the market is a bigger ballgame. For any technology advancements from bench to bedside, some classical bottlenecks are well known. These include validation with a gold standard, scalability, market dynamics, affordability, commercial performances, etc., and acceptability in clinical practice. Some of these challenges for AI integration in the medical sector are well-discussed by a few national medical academies. For example, the United Kingdom Royal Society and Academy of Medical Sciences, in its 2019 report, highlighted a lack of adequate computing power and challenges in data accessibility, sharing, and use with poor ‘digital maturity’ in the United Kingdom (The Royal Society and Academy of Medical Sciences, 2019). Indian National Academy of Medical Sciences summarized six challenges. In addition to the opinions of the United Kingdom, the Indian academy noted a lack of skilled human resources, unstructured governance and regulations, poor funding ecosystem, and few ethical and societal concerns while introducing AI in the medical sector (National Academy of Medical Sciences, 2023). Interestingly, in terms of IP protection, AI creates an additional layer of unresolved bottlenecks for the research and commercial sectors. However, hardly any Medical Academies have stressed this. Indian Medical Academy noted an ‘unattractive IP regime’ as impediment toward full utilization of AI in healthcare yet made no recommendations toward course correction.

Intellect by itself is a virtue but not a property. However, it became a property when there was a need to protect one’s creativity amid competition and commercial implications. Every legal instrument that is the law of the land essentially constructs a barrier around scientific practices to keep the harm away, ensure peaceful use, maintain ethics, and protect the developer’s rights. Wherein safety, security, and ethics create a harmonious R&D ecosystem for progressive S&T, the IP incentivizes and recognizes a developer for their innovation. IP is not about risk/ threat reduction; instead, it creates momentum in the S&T community to engage and invest more in innovative endeavors, knowing their work will be rewarded. For an academician, it could be a patent or copyright that helps in career growth and possibilities of technology commercialization either by self or through technology transfer. For a company, the patent mainly grants a competitive edge in securing trade advantages and gaining substantial financial benefits over its competitors. As such, innovators of any origin sought a stable IP regime with legislative clarity, simplified transactions, and rapid resolutions against infringement issues. In its current state, AI is just the opposite of those. Unfortunately, there is no readily available central database that tracks all AI patent infringement cases globally.

However, given these limitations, it is safe to say that the total number of AI patent infringement cases may be a hundred globally and likely continue to grow. Two notable cases exist in the biomedical sector. The first is the AliveCor vs. Apple case in 2022, where the former claimed that Apple infringed three of its patents related to AI-based cardiac diagnostic technology in the ECG feature of the Apple Watch. While the United States Patent Office ruled in favor of Apple, the United States International Trade Commission (ITC) held Apple guilty of infringing the patents (DAIC, 2023). The second example is the Philips vs. Masimo case of 2014. Masimo sued Philips by alleging infringement of its patents on pulse oximetry technology that uses AI-based signal processing techniques for measuring blood oxygen levels. United States court returned a verdict in favor of Masimo and awarded $466,774,783 in damages (Masimo Masimo, 2015).

Here, we have reviewed the legal instruments of a few nations (Table 1). From this, it is clear that IP policy and laws addressing AI applications, in general, are evolving. The existing IPR laws were penned down to protect and preserve human intelligence, but AI was never thought of. As such, while receiving the AI-based innovation application, patent office’s interpret the laws in the best possible way to give maximum freedom to accommodate AI. The most pressing question has been the ‘Who is the author’? Most Western nations still prefer to delegate authorship to humans only. In contrast, countries like China and India have given co-authorship to AI. It has often landed in disputes and resulted in decisions through court interventions. It resulted in unwanted delays in innovation development and its commercialization.

**Table 1:** Comparative table on the differences in IP laws across jurisdictions and its impact of artificial intelligence (AI).

Country	Act/Law	Implementing agency	Year enacted	Focus area	Key provisions	Scope/Impact on AI
United States	Patent Act (Title 35 of the United States Code)	United States Patent and Trademark Office (USPTO)	1790 and amendments thereafter	Patent	Patentability requirements, utility model protection, design patents, patent infringement	Patentability of AI-related inventions
	United States Copyright Act of 1976	United States Copyright Office (USCO)	1976 and amendments thereafter	Copyright	Copyright ownership, fair use, duration, infringement	Protects AI-generated works (with potential challenges), software code, and user interfaces.
	Health Insurance Portability and Accountability Act (HIPAA)	Department of Health and Human Services (HHS)	1996 and amendments thereafter	Data/ Health Information	Privacy and security of medical records	Regulate the use and disclosure of patient data for AI development and applications.
	National Artificial Intelligence Initiative (NAII) Act 2021	Multiple Federal Agencies (coordinated by the White House Office of Science and Technology Policy)	2021	AI	Coordination of AI research and development through Federal agency collaboration, AI research institutes, standards development	Supports AI innovation by fostering research and development, but does not directly address intellectual property.
	Future of AI Innovation (FAII) Act of 2024	Multiple Federal Agencies (likely coordinated by the White House Office of Science and Technology Policy)	2024	AI	Promoting AI innovation and development, Standards, metrics, research funding, industry support	Directly impacts AI by establishing standards, promoting innovation, and potentially influencing patent and copyright law.
European Union	European Union Patent Law	European Patent Office (EPO)	1977	Patent	Granting and enforcing patents, patent validity and infringement	Patentability of AI-related inventions
	European Union Copyright Law	National copyright offices, EUIPO (for certain aspects)	2001	Copyright	Copyright ownership, fair use, term, infringement	Covers AI-generated content, software code, and databases used for AI training, raising questions about ownership and infringement.
	General Data Protection Regulation (GDPR)	National supervisory authorities	2018	Data/ Health Information	Data subject rights, data processing principles, data breaches, privacy	Impacts AI by regulating the processing of personal data for training and operation of AI systems.
	New European Innovation Agenda (NEIA)	European Commission	2021	Innovation	Fostering innovation, digital transformation, Investment, skills, regulation, international cooperation	Supports AI development by creating a conducive environment for research, development, and deployment.
	EU’s Data Governance Act (DGA)	National supervisory authorities	2023	Data	Data intermediaries, data spaces, data sharing agreements	Facilitates data access for AI development by creating legal frameworks for data sharing and reuse.
	Artificial Intelligence (AI) Act	European Commission	2024	AI	Risk-based approach, transparency, human oversight	Establishes a framework for AI development and use, including provisions on data, transparency, and liability.
India	India’s Copyright Act, 1957	Copyright Office, India	1957 and amendments thereafter	Copyright	Copyright ownership, infringement, fair use	Protects software code, AI-generated content (with challenges), and databases used for AI training.
	Information Technology (IT) Act, 2000	Ministry of Electronics and Information Technology (MeitY)	2000 and amendments thereafter	Data	Digital signature, e-commerce, cybercrime, data protection (initial provisions)	Provides a legal framework for digital activities, including AI, but with limited specific provisions for AI.
	Patents (Amendment) Act, 2005	Indian Patent Office (IPO)	2005	Patent	Amended patent law to align with TRIPS agreement, introduced product patents in certain areas	Patentability of AI-related inventions
	Information Technology (Reasonable Security Practices and Procedures and Sensitive Personal Data or Information) Rules, 2011	Ministry of Electronics and Information Technology (MeitY)	2011	Data/ Health Information	Security practices, sensitive personal data	Impacts AI by setting data protection standards, relevant for AI systems handling personal data.
	Digital Personal Data Protection (DPDP) Act, 2023	Ministry of Electronics and Information Technology (MeitY)	2023	Data/ Health Information	Comprehensive data protection framework, data processing, cross-border data transfer	Regulate the processing of personal data for AI development and deployment.
China	Patent Law of the People’s Republic of China	State Intellectual Property Office (SIPO)	1984 and amendments thereafter	Patent	Patentability criteria, patent infringement, compulsory licensing	Patentability of AI-related inventions
	China’s Copyright Law	National Copyright Administration (NCA)	1990 and amendments thereafter	Copyright	Copyright ownership, infringement, fair use	Protects software code, AI-generated content (with challenges), and databases used for AI training.
	Cybersecurity Law (2017)	Cyberspace Administration of China (CAC)	2017	Data	Network security, data localization, critical information infrastructure, personal data protection	Impacts AI development by imposing security requirements, data localization, and restrictions on data transfer.
	Data Security Law	Cyberspace Administration of China (CAC)	2020	Data	Classification of data, data protection measures, cross-border data transfer	Impacts AI development by setting data security standards and restrictions on data usage.
	Personal Information Protection Law (PIPL) (2021)	Cyberspace Administration of China (CAC)	2021	Data/ Health Information	Data collection, processing, cross-border data transfer, security	Regulating the processing of personal data for AI development and deployment.

Considering the immense addition of value that AI can do in the biomedical sector, we recommend the sector-specific bottom-up approach from practice to policy-level discussion for formulating a predictable and stable IP regime. Only the biomedical sector-specific discussion can specifically identify supply–demand, user-usage, and necessity-usability issues unique to it and even vary across nations, mainly depending on economic status. A collaborative approach involving stakeholders across the biomedical ecosystem will be required. It includes policymakers, clinicians, researchers, industry leaders, legal professionals, and patient advocacy groups. In this, bridging developers (researchers, industry groups) and end users like clinicians and patient advocacy groups is essential to avoid the known ‘valley of death’ scenario that has traditionally troubled the translation and adoption of biomedical innovations. The other arm of collaboration is to discuss and implement standardized data-sharing agreements while addressing data security and privacy concerns in digital space. Only through these actions, it is possible to develop solutions that foster innovation, protect intellectual property rights, and ultimately benefit patients worldwide.

Although we see few national developments in adopting the National AI framework, it is not enough. Most of these frameworks have neither made a strong case for IPs nor actionable strategies to deal with IP bottlenecks. Without transparent deliberations, WIPO has taken the lead since 2019. As part of its Second Session of Conversation on Intellectual Property and Artificial Intelligence, WIPO issued a draft paper with pressing questions covering patents, copyright, data, designs, technology gaps, capacity building, and accountability for IP administrative decisions (WIPO, 2019). WIPO sought responses from global stakeholders across Government, Non-Government organizations, industries, academia, etc. With around 250 submissions against the call, WIPO issued a revised draft in 2020. The Third Session discussed it in detail (WIPO, 2020). At the latest, WIPO completed its sixth conversation session with a deep dive into the AI and IP issues with information collected from past conversations (WIPO, 2022). WIPO mentioned addressing the AI inventorship in 2023, which could assist nations in creating their legal baseline. Although we expect that WIPO will release this soon, national policymakers may well self-explore the outcome of these conversations to start working on an informed IP regime for AI.

A straightforward option could be exploring entirely new IP protection mechanisms for AI-based innovations instead of playing around with existing IP laws and issuing multiple amendments to accommodate AI. There is quite an intense debate about whether the fundamental definition of intellect varies between human and artificial intelligence. As such, rules specific to humans may not apply to AI; instead, new sets of rules seemed rational. Adopting new laws can immediately place the much-desired informed and stable IP regime and be a lifesaver for AI developers. This strategy can also help establish clear guidelines for inventorship and attributing IP rights. It will incentivize innovation and ensure fair compensation for developers. Contrary to these benefits, introducing new legislation requires Parliament to pass and approve, especially in large democracies like the United States and India (Stepan, 2005). The process can take an indefinite time and ultimately compromise the fundamental need for this reform. However, the report suggests that India is planning for a category in the IPR regime and, hopefully, can guide the world in this direction.

Only the future can tell how the IP dilemma will be explicitly addressed for AI-based innovation in the biomedical sector. However, given the drastic rise of AI and its transformative protection in the biomedical sector across research and healthcare, we believe that nations will fast-track strategies to implement a stable IP regime.
